# Alterations of the Gut Microbiome in Hypertension

**DOI:** 10.3389/fcimb.2017.00381

**Published:** 2017-08-24

**Authors:** Qiulong Yan, Yifang Gu, Xiangchun Li, Wei Yang, Liqiu Jia, Changming Chen, Xiuyan Han, Yukun Huang, Lizhe Zhao, Peng Li, Zhiwei Fang, Junpeng Zhou, Xiuru Guan, Yanchun Ding, Shaopeng Wang, Muhammad Khan, Yi Xin, Shenghui Li, Yufang Ma

**Affiliations:** ^1^Department of Biochemistry and Molecular Biology, Dalian Medical University Dalian, China; ^2^Department of Microbiology, Dalian Medical University Dalian, China; ^3^Shenzhen Puensum Genetech Institute Shenzhen, China; ^4^Beijing Genomics Institute Shenzhen, China; ^5^Department of Laboratory Diagnostics, The First Affiliated Hospital of Harbin Medical University Harbin, China; ^6^Department of Cardiology V, The Second Affiliated Hospital of Dalian Medical University Dalian, China; ^7^Department of Cardiology, The First Affiliated Hospital of Dalian Medical University Dalian, China; ^8^College of Basic Medical Sciences, Dalian Medical University Dalian, China; ^9^Department of Biotechnology, Dalian Medical University Dalian, China

**Keywords:** hypertension, gut microbiome, microbial dysbiosis, metagenome-wide association study

## Abstract

**Introduction:** Human gut microbiota is believed to be directly or indirectly involved in cardiovascular diseases and hypertension. However, the identification and functional status of the hypertension-related gut microbe(s) have not yet been surveyed in a comprehensive manner.

**Methods:** Here we characterized the gut microbiome in hypertension status by comparing fecal samples of 60 patients with primary hypertension and 60 gender-, age-, and body weight-matched healthy controls based on whole-metagenome shotgun sequencing.

**Results:** Hypertension implicated a remarkable gut dysbiosis with significant reduction in within-sample diversity and shift in microbial composition. Metagenome-wide association study (MGWAS) revealed 53,953 microbial genes that differ in distribution between the patients and healthy controls (false discovery rate, 0.05) and can be grouped into 68 clusters representing bacterial species. Opportunistic pathogenic taxa, such as, *Klebsiella* spp., *Streptococcus* spp., and *Parabacteroides merdae* were frequently distributed in hypertensive gut microbiome, whereas the short-chain fatty acid producer, such as, *Roseburia* spp. and *Faecalibacterium prausnitzii*, were higher in controls. The number of hypertension-associated species also showed stronger correlation to the severity of disease. Functionally, the hypertensive gut microbiome exhibited higher membrane transport, lipopolysaccharide biosynthesis and steroid degradation, while in controls the metabolism of amino acid, cofactors and vitamins was found to be higher. We further provided the microbial markers for disease discrimination and achieved an area under the receiver operator characteristic curve (AUC) of 0.78, demonstrating the potential of gut microbiota in prediction of hypertension.

**Conclusion:** These findings represent specific alterations in microbial diversity, genes, species and functions of the hypertensive gut microbiome. Further studies on the causality relationship between hypertension and gut microbiota will offer new prospects for treating and preventing the hypertension and its associated diseases.

## Introduction

Hypertension is a global public health problem. In 2010, about 31% of the world's population has been estimated to suffer from hypertension and over 1 billon of this population is living in low- and middle- income countries(Mittal and Singh, 2010; Mills et al., 2016). Hypertension is one of the major risk factors for cardiovascular diseases, such as, stroke and heart failure (Lim et al., 2012; Faraco and Iadecola, 2013). Moreover, it is believed to be one of the most common comorbidities associated with chronic renal disease (Lash et al., 2009), obesity and type 2 diabetes (Landsberg and Molitch, 2004; Kotchen, 2010). Presently, genome-wide association studies (GWAS) have identified a series of genetic loci and pathways associated with blood pressure (Xu et al., 2015; Liu et al., 2016). The environmental factors, such as, dietary salt intake, alcohol consumption and lack of exercise, are also linked to the occurrence of hypertension (Fuchs et al., 2001; Karppanen and Mervaala, 2006). Recent practice of metabolomics also identified new pathogenic pathways involved in blood pressure regulation (Menni et al., 2015; Galla et al., 2017). Nevertheless, due to the complexity and heterogeneity of hypertension, identification of the causes of this disease is still challenging.

Recent studies have demonstrated that the gut microflora plays an essential role in development of cardiovascular diseases, via metabolizing dietary choline, phosphatidylcholine and L-carnitine to produce trimethylamine (TMA), which is further oxidized into TMA N-oxide (TMAO, a metabolite that enhances atherosclerosis; Wang et al., 2011; Koeth et al., 2013; Tang et al., 2013). Even though the direct link between hypertension and TMAO has not been established currently, TMAO's role to prolong the hypertensive effect of angiotensin II were reported (Ufnal et al., 2014). Inhibition of gut microbiota-mediated TMAO production may serve as a potential therapeutic approach for the treatment of cardiometabolic diseases (Wang Z. et al., 2015). These findings suggest an intricate and predictable correlation between hypertension and gut microbiota. To validate this, a recent study based on metagenomic analyses of the fecal samples of 41 healthy controls, 56 pre-hypertension subjects, and 99 hypertension individuals described a novel causal role of aberrant gut microbiota in contributing to the pathogenesis of hypertension, and emphasized the significance of early intervention for pre-hypertension (Li et al., 2017). Moreover, rat experiments have linked gut microbial dysbiosis with hypertension (Mell et al., 2015; Yang et al., 2015; Adnan et al., 2017; Santisteban et al., 2017). The causal role of gut microbiome in obstructive sleep apnea-induced hypertension have been reported (Durgan et al., 2016). Here, to investigate the alteration of the human gut microbiome underlying hypertension, we compared the microbial composition of fecal samples obtained from 60 patients with primary hypertension and 60 healthy counterparts of Chinese origin. We used quantitative metagenomic analysis to identify genic, microbial, and functional characteristics underlying hypertension.

## Methods

### Subjects and sample collection

Sixty primary hypertensive patients (current blood pressure ≥140/90 mm Hg) and sixty gender-, age-, and body weight-matched healthy controls (current blood pressure ≤ 120/80 mm Hg) were recruited for this study. Other than systolic blood pressure (SBP) and diastolic blood pressure (DBP), the other clinical parameters have no significant differences in the two groups of populations, except for triglyceride (TG). The characteristics of the subjects are summarized in Table 1, and detailed information is given in Table S1. Subjects were excluded if they had symptoms of respiratory infection or digestive tract disease, or if they were treated with antibiotics or anti-inflammatory agents in recent 2 months before sampling. Subjects with hypertension or severe cardiovascular diseases (such as, coronary artery disease or stroke) history in previous 5 years were also excluded from healthy controls. Fresh fecal samples were collected from each subject and were stored at a −80°C freezer immediately.

**Table 1:** Characteristics of subjects.

	**Case (*n* = 60)**	**Control (*n* = 60)**	***P*-value**
Gender (F/M)	25/35	28/32	0.713
Age, y	57.0 ± 9.6	56.0 ± 8.6	0.523
BMI, kg/m^2^	23.5 ± 2.9	23.4 ± 2.6	0.854
SBP, mm Hg	165 ± 20	111 ± 6	<0.001
DBP, mm Hg	101 ± 11	71 ± 7	<0.001
FGB, mmol/L	6.37 ± 2.39	6.19 ± 2.09	0.649
HDL, mmol/L	1.15 ± 0.24	1.23 ± 0.29	0.112
LDL, mmol/L	3.08 ± 0.76	3.04 ± 0.71	0.752
TG, mmol/L	1.87 ± 0.85	1.52 ± 0.69	<0.05
TC, mmol/L	5.02 ± 0.97	5.07 ± 0.97	0.791
Smoke	31.7%	40.0%	0.447

*The data for age, BMI, SBP, DBP, FGB, HDL, LDL, TG, and TC were presented as mean ± SD. P-values for gender and smoke were calculated by Fisher's exact test. P-values for age, BMI, SBP, DBP, FGB, HDL, LDL, TG, and TC were calculated using Student's t-test. BMI, body mass index; SBP, systolic blood pressure; DBP, diastolic blood pressure; HDL, high density lipoprotein; LDL, low density lipoprotein; TG, total triglyceride; and TG, total cholesterol*.

### Ethics statement

This study received approval from the ethics committee of The First Affiliated Hospital of Harbin Medical University, and written informed consent was obtained from each participant. The methods were carried out in accordance with the approved guidelines.

### DNA preparation and sequencing

Genomic DNA was extracted from all samples according to a modified protocol provided in the QIAamp DNA mini kit (Qiagen, Manchester, UK; Yan et al., 2016). Briefly, ASL buffer (1.4 ml) was added to 220 mg of fecal sample and the pellets were homogenized in a 2 ml screw cap tubes (Axygen) by vortex. The suspension was incubated at 95°C for 5 min to lyse bacterial cells. After centrifugation (13,000 × g, 1 min) and incubation with an InhibitEx tablet, the supernatant was treated with 15 μl proteinase K and 200 μl Buffer AL at 70°C for 10 min. The extracted DNA was dissolved in 100 μl sterile water. Paired-end DNA libraries (insert size 350 bp, read length 150 bp) were constructed according to the manufacturer's instructions (Illumina, USA). Whole-metagenome shotgun sequencing was performed on the Illumina HiSeq3000 platform. Further methodological detail is available in the Supplementary Methods.

### Bioinformatic analysis

#### Quantification of metagenomic genes and species

High-quality reads from each sample were aligned to the integrated non-redundant human gut gene catalog (IGC; Li et al., 2014) using SOAP2 (Li et al., 2009; >90% similarity). The relative abundance of a gene in a sample was estimated by dividing the number of reads uniquely mapped to that gene by the length of gene region and by the total number of reads from the sample. We also aligned the sequencing reads against the available microbial genomes (bacteria, archaea, and virus) from the National Center for Biotechnology Information (NCBI) database and generated the taxonomic compositions (i.e., phylum and genus composition) for all samples.

#### Alpha diversity

The gene count (Le Chatelier et al., 2013) of a metagenomic sample were calculated based on their mapped reads number on the gene catalog (to eliminate the influence of sequencing amount fluctuation, 10 million reads were randomly extracted from each sample for mapping). The Shannon index (within-sample diversity) was calculated based on the gene relative abundance profiles, using the method described previously (Qin et al., 2012).

#### Functional annotation and profiling

The Kyoto Encyclopedia of Genes and Genomes (KEGG, downloaded Jan-2016) database (Kanehisa et al., 2014) was used for functional annotation of genes. Amino acid sequences were searched against the databases using USEARCH v8.1 (Edgar, 2010) with a minimum similarity of 30%. Each gene was assigned a KEGG ortholog (KO) based on the best hit protein. The abundance profiles of KO were calculated by summing the relative abundance of its genes. The choline-trimethylamine lyase (*cutC*, KO: K20038; Craciun and Balskus, 2012) was used to evaluate the gut microbiota-mediated TMA production in subjects, and the short-chain fatty acid (SCFA)-producing enzymes were represented by acetyl-CoA decarbonylase/synthase (K00193, K00194, K00197, K14138, which are key enzymes of the acetate biosynthesis pathways: KEGG modules M00377 and M00422; Koh et al., 2016), propionyl-CoA:succinate-CoA transferase (Reichardt et al., 2014), butyryl-CoA:acetate CoA-transferase (K01034, K01035), and butyrate kinase (K00929) (Pryde et al., 2002; Louis et al., 2010).

#### Metagenome-wide association study

We used the metagenome-wide association study (MGWAS) method to identify gene markers that showed significant abundance differences between hypertensive patients and control subjects. The MGWAS was performed using the methodology developed by Qin et al. (2012). Co-abundance genes were clustered into metagenomic linkage groups (MLGs) based on the previous methods (Qin et al., 2012). Taxonomic assignment and abundance profiling of the MLGs were performed according to the taxonomy and the relative abundance of their constituent genes (see Supplementary Methods for detail). MLGs were considered to be interacted if absolute value of Spearman's correlation coefficient between them is greater than 0.4, and the co-occurrence network of MLGs was visualized by Cytoscape (Shannon et al., 2003).

### Statistical analyses

Statistical analyses were implemented using the R platform. Distance-based redundancy analysis (dbRDA) was performed on normalized taxa abundance matrices with R *vegan* package (Dixon, 2003) according to Bray-Curtis distance, then visualized with R *ggplot2* package. Random forest models were trained with R *randomForest* package (10,000 trees) to predict hypertension status according to MLG abundance profiles. The performance of the predictive model was evaluated with cross-validation error. Receiver operator characteristic (ROC) analysis was performed using R *pROC* package. *P*-value < 0.05 was considered statistical significance, and the *q*-value was calculated to evaluate the false discovery rate (FDR) for correction of multiple comparisons.

### Data availability

The raw whole-metagenomic shotgun sequencing data acquired in this study have been deposited to the European Bioinformatics Institute (EBI) database under the accession code ERP023883.

## Results

### Comparison of the gut microbiota between hypertensive patients and controls

**Figure 1:**
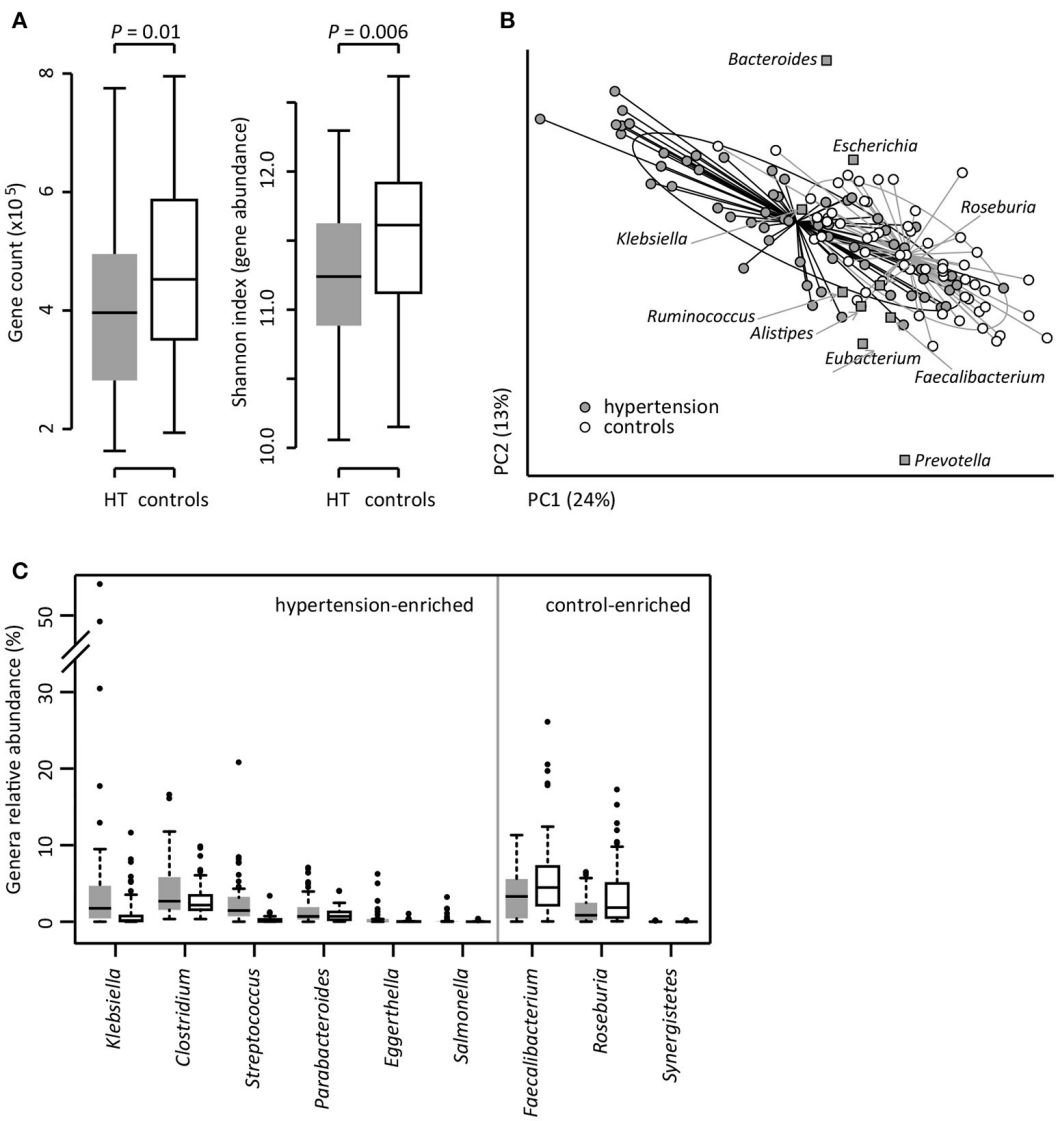
Difference of gut microbial community between hypertensive patients and controls. **(A)**, Difference of alpha diversity between hypertensive (HT) patients and controls. **(B)**, dbRDA based on the Bray-Curtis distances between microbial genera, revealing a hypertensive microbial dysbiosis which overlaps only in part with taxonomic composition in patients and controls. The first two principle components and the ratio of variance contributed is shown. Genera (square) as the main contributors are plotted by their loadings in these two components. Lines connect samples in the same group, and circles cover samples near the center of gravity for each group. **(C)**, Boxplot shows the significantly different genera between patients and controls. Genera with *q* < 0.05 (Mann-Whitney *U*-test corrected by FDR) are shown. Only the genera with average relative abundances greater than 0.05% of total abundance in all samples are shown for clarity. Gray and white boxes represent the patients and controls, respectively. For A and C, the boxes represent the interquartile range (IQR) between first and third quartiles and the line inside represents the median. The whiskers denote the lowest and highest values within 1.5 times IQR from the first and third quartiles, respectively. The dots represent outliers beyond the whiskers.

To investigate the gut microbial composition of 60 hypertensive patients and 60 healthy controls, we obtained 652.9 Gbp high-quality data (5.4 ± 1.1 Gbp per sample) via whole-metagenome shotgun sequencing on their fecal samples. When we quantified the microbial (alpha) diversity within each subject, the patients showed significantly lower gene count and Shannon index compared with the controls (Figure 1A). Multivariate analysis based on Bray-Curtis distance between microbial genera revealed remarkable differences between patients and controls (Figure 1B). At the phylum level, patients had higher levels of Proteobacteria (*p* < 0.01), but fewer Actinobacteria (*p* = 0.02). At the genus level, *Klebsiella, Clostridium, Streptococcus, Parabacteroides, Eggerthella*, and *Salmonella* were frequently distributed in hypertensive gut compared to normal controls while *Faecalibacterium, Roseburia*, and *Synergistetes* were found to be higher in control group compared to hypertensive patients (Figure 1C). These findings demonstrated considerable gut microbial dysbiosis in hypertensive patients.

### Identification of hypertension-associated markers from gut microbiome

**Figure 2:**
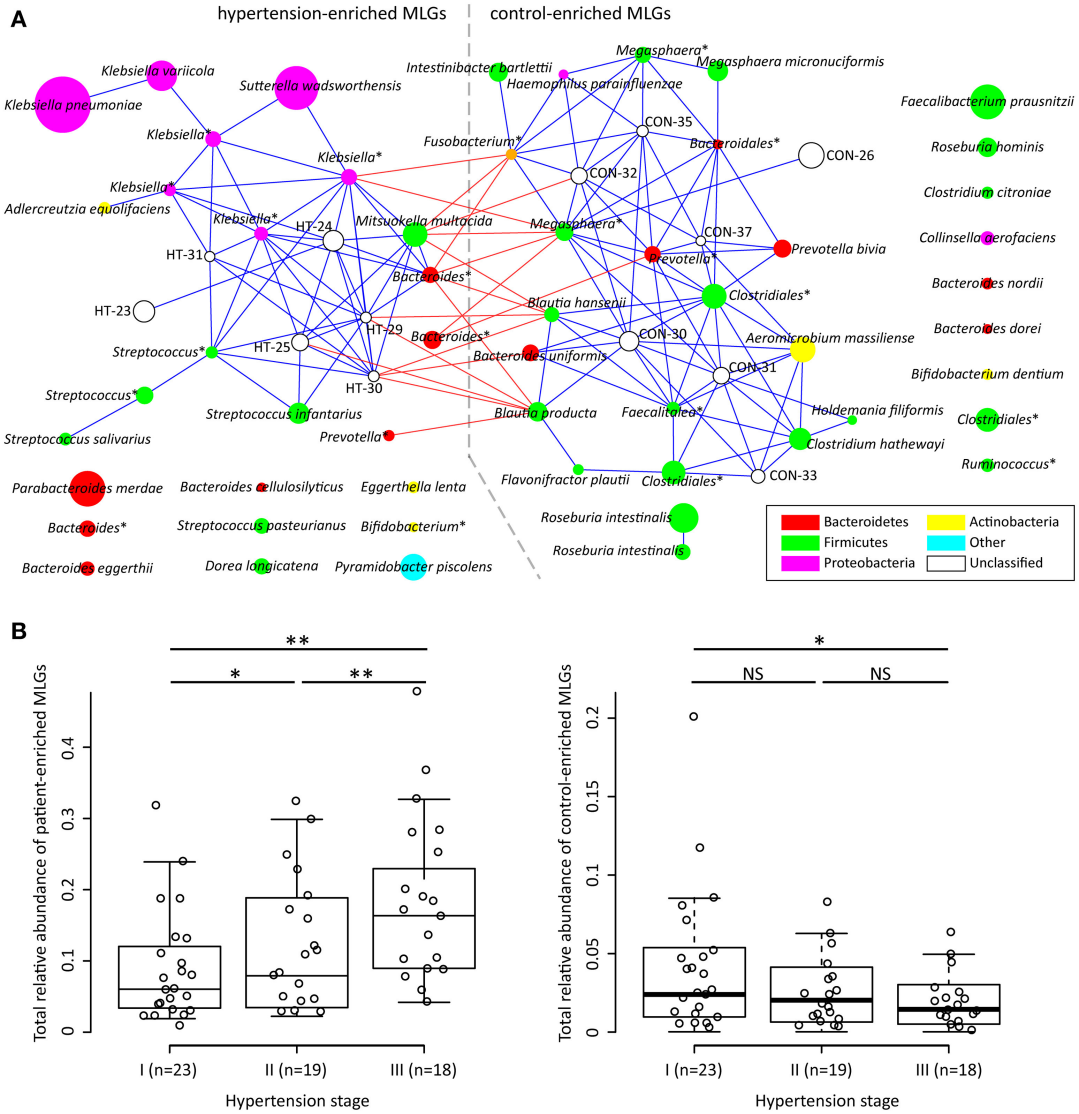
Characterization and interconnection of hypertension-associated MLGs. **(A)** Co-occurrence network shows the interconnection of the hypertension- and control-enriched MLGs. Nodes depict MLGs with their ID or taxonomic assignment (unclassified MLGs under genus or higher taxonomy rank are marked by “^*^”) displayed in the center. The size of the nodes indicates the number of gene within the MLG. Connecting lines represent Spearman correlation coefficient ρ > 0.40 (represented by blue line) or < −0.40 (represented by red line). **(B)**, Correlation of gross abundance of hypertension- and control-enriched MLGs with hypertension stage. NS, not significant; ^*^, *q* < 0.05; ^**^, *q* < 0.01; Mann-Whitney *U*-test corrected by FDR.

To explore signatures of the gut microbiome in hypertensive patients and controls, we integrated the sequencing data into an existing gut microbial reference gene catalog to obtain a set of 5.3 million genes, which allowed for saturation mapping of the reads (80.3%). Using the MGWAS methods, we identified 53,953 genes that showed a significant difference between two groups (FDR corrected *q* < 0.05). Approximately, 69% of these genes were clustered into 68 metagenome linkage groups (MLGs, Table S2), that allowed to species level description for the microbiome differences. Thirty-one MLGs were higher in patients while 37 in controls. Consistent with the genus level observations, MLGs of *Klebsiella* (mainly consisting of *K. pneumoniae* and *K. variicola*), *Streptococcus* (*S. infantarius, S. pasteurianus* and *S. salivarius*), and *Parabacteroides merdae* were found to be higher in hypertensive samples, whereas MLGs of *Roseburia* (mainly consisting of *R. intestinalis* and *R. hominis*) and *Faecalibacterium prausnitzii* were higher in controls. Moreover, the MLGs enriched in hypertensive patients also contain several *Bacteroides* spp. (including *B. eggerthii* and *B. cellulosilyticus*), *Sutterella wadsworthensis* and *Pyramidobacter piscolens*, and the MLGs enriched in controls include several other *Bacteroides* spp. (including *B. uniformis, B. nordii* and *B. dorei*), *Megasphaera* spp. (*M. micronuciformis*), and *Aeromicrobium massiliense*. A co-occurrence network on these MLGs revealed a large number of interconnections within hypertension-enriched and control-enriched MLGs (Figure 2A), as well as some MLGs derived from two groups negatively correlated. This result suggested that the MLGs did not occur independently and interacted with the taxa in its environment.

We next found that the gross abundances of hypertension- and control-enriched MLGs are correlated to the severity of hypertension (Figure 2B), suggesting that the bacterial relative abundance of these MLGs could be related to the development and disease progress of hypertension.

### Functional characterization of gut microbiota

**Figure 3:**
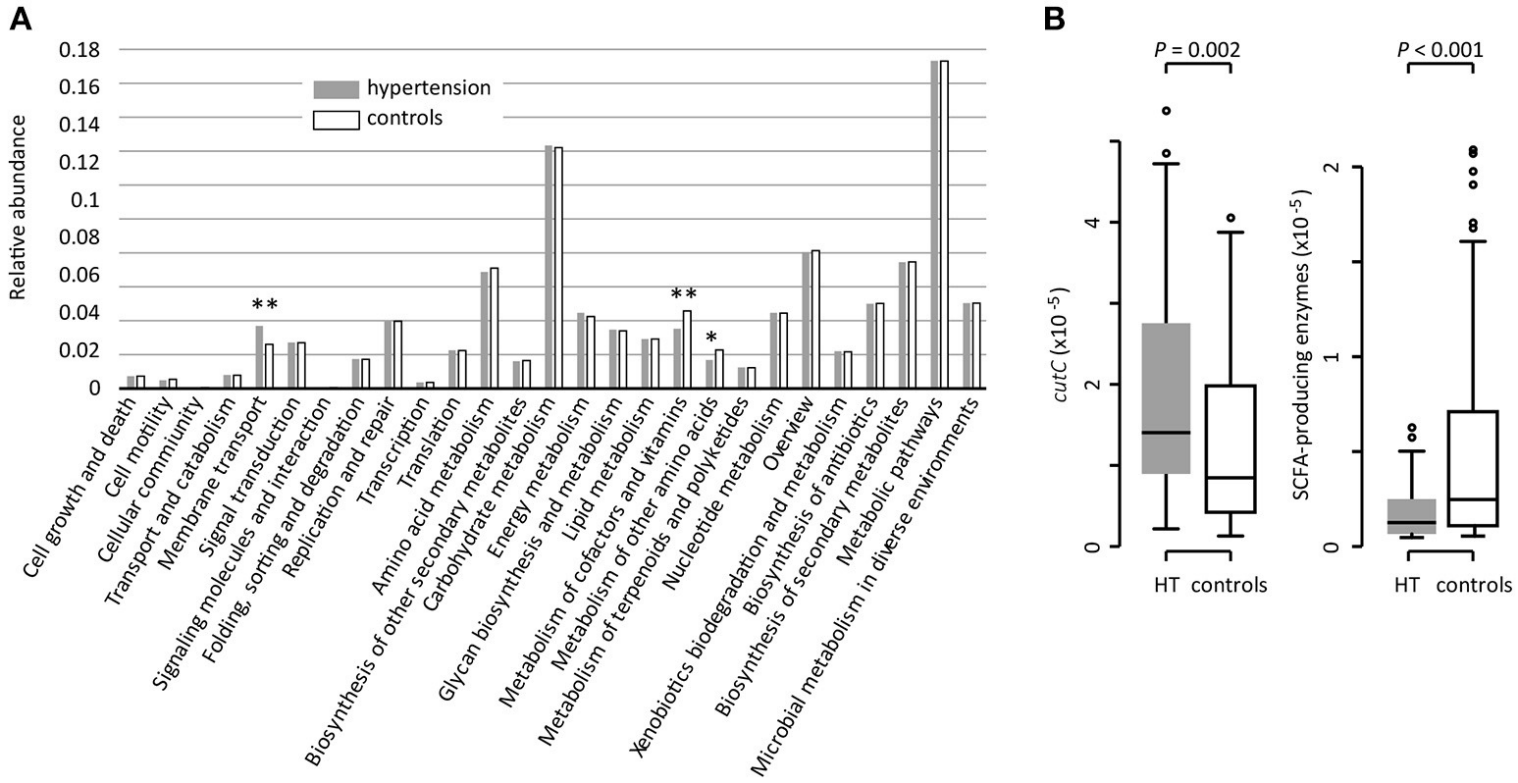
Functional comparison of the gut microbiomes between hypertensive patients and healthy controls. **(A)**, Distributions of relative abundances of KEGG pathway categories in hypertensive patients and controls. ^*^, *q* < 0.05; ^**^, *q* < 0.01; Mann-Whitney *U*-test corrected by FDR. **(B)**, Difference of the relative abundance of *cutC* (TMA-producing) and SCFA-producing enzymes between hypertensive (HT) patients and controls.

Based on the KEGG pathway comparison, we revealed that the hypertensive gut microbiomes were more abundant in membrane transport, lipopolysaccharide (LPS) biosynthesis, and steroid degradation (Figure 3A and Table S3), while the controls were enriched in metabolism of “other amino acids,” cofactors and vitamins (including folate biosynthesis and metabolism, riboflavin metabolism, and ubiquinone biosynthesis). In addition, the gut microbial enzymes involved in TMA production were enriched in the hypertensive patients compared to controls, whereas the SCFA-producing enzymes were depleted (Figure 3B).

### Gut microbiota-based classification of hypertension

**Figure 4:**
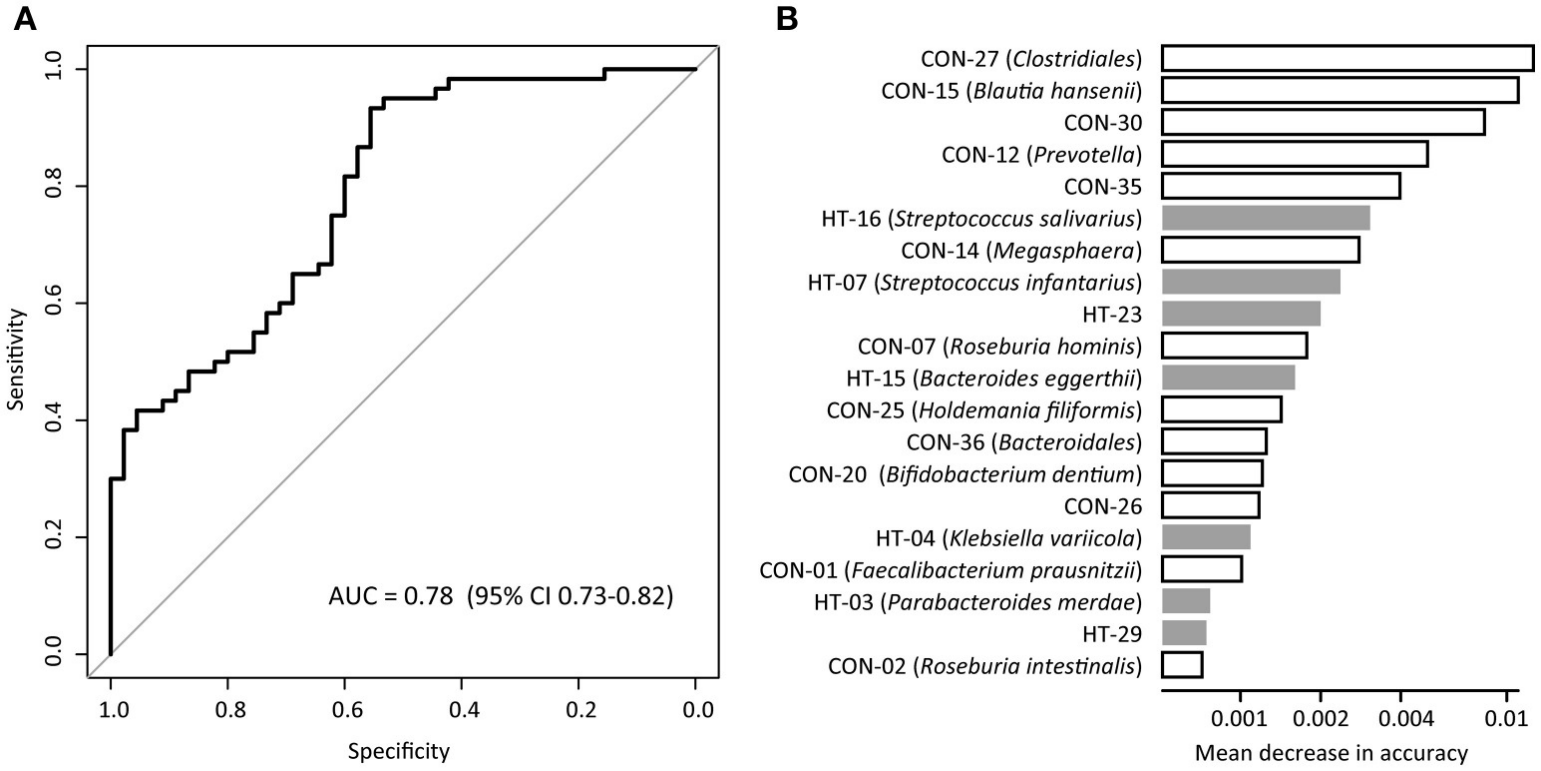
Classification of hypertension status by the abundances of MLGs. **(A)**, ROC analysis for classification of hypertensive status by MLGs, assessed by AUC. **(B)**, The 20 most discriminant MLGs in the model classifying hypertensive patients and healthy controls. The bar lengths indicate the importance of the variable, and colors represent enrichment in patients (black) or controls (white).

We evaluated the performance of gut microbiota composition to identify hypertension status in the MLG profiles using the Random Forest model, and obtained the discriminatory power of the area under the ROC curve (AUC) of 0.78 (95% CI 0.73–0.82; Figure 4A). Several control-enriched MLGs (including *Clostridiales, Blautia hansenii, Megasphaera*) and two hypertension-enriched members of *Streptococcus* (*S. salivarius* and *S. infantarius*) featured the highest score for the discrimination of hypertensive patients and healthy controls (Figure 4B).

## Discussion

To identify and analyze the differences of the gut microbiota in hypertension, we characterized the genic, microbial, and functional repertoire of the microbiomes of 60 hypertensive patients and 60 gender-, age-, and body weight-matched controls. Our study strengthened previous metagenomic study on gut microbiome of hypertension (Li et al., 2017) by adding more information. Furthermore, we observed significant differences in microbial community dysbiosis, taxonomic shifts, and functional changes between hypertensive- and control-gut microbiome.

Previous studies showed that the gut microbes participate in choline and phosphatidylcholine metabolism to form circulating and urinary TMAO, while high levels of plasma TMAO promote accelerated atherosclerosis and increase the risk of cardiovascular disorders (Tang et al., 2013; Wang Z. et al., 2015). The choline utilization (*cutC*) gene, a critical gene that coverts the choline to trimethylamine, was identified in a variety of human gut commensals belonging to Firmicutes, Proteobacteria, and Actinobacteria (Craciun and Balskus, 2012). Notably, several genera such as, *Klebsiella, Clostridium*, and *Streptococcus*, which are highly distributed in hypertensive patients are choline degraders (Hakenbeck et al., 2009; Craciun and Balskus, 2012; Kalnins et al., 2015). Functional analysis also showed that the abundance of *cutC* gene was enriched in the gut microbiota of the hypertensive patients. These findings suggested that the dietary choline intake and TMAO production via gut microflora would be a probable pathway for hypertensive pathogenesis.

*Klebsiella*, is a pathogen routinely found in human gut that causes pneumonia, diarrhea, and urinary tract infection. The distribution of *Klebsiella* was found to be significantly higher in hypertensive patients compared to healthy controls as evident from Figure 1C. Overgrowth of *Klebsiella* usually foreshadows gut flora dysbiosis, which leads to a variety of serious chronic disease, such as, colitis (Garrett et al., 2010), Crohn's disease and ankylosing spondylitis (Ebringer et al., 2007). The present study demonstrates that *Klebsiella* species which are highly distributed in hypertensive patients are *K. pneumoniae* (the main component of *Klebsiella* that associated with nosocomial infection and multiple diseases), *K. variicola* [a human and animal opportunistic pathogen that is associated with bovine mastitis (Brisse and Duijkeren, 2005)], and four unclassified MLGs. Based on these information, however, the potential correlation between *Klebsiella* and hypertension is still unclear.

*Streptococcus*, the dominant species of human oral microbiome (Wade, 2013) that causes upper respiratory tract infection, were also found highly distributed in gut microbiota of hypertensive patients as compared to the controls. Gut streptococci is also associated with diseases, such as, inflammatory bowel disease (Conte et al., 2006) and liver cirrhosis (Qin et al., 2014). It has been reported previously that oral cavity and/or gut might be the source of streptococci found in the majority of atherosclerotic plaque microbiota (Koren et al., 2011). These findings suggest that possible correlation of gut streptococci in hypertension.

*F. prausnitzii* and *Roseburia* spp., which were abundantly distributed in controls compared to hypertensive patients, were also distributed abundantly in the healthy control microbiomes of many chronic diseases, including type 2 diabetes (Qin et al., 2012), liver cirrhosis (Qin et al., 2014), Crohn's disease (Gevers et al., 2014), and ulcerative colitis (Machiels et al., 2014). *F. prausnitzii* and *Roseburia* (both *R. intestinalis* and *R. hominis*) are the major SCFA producer in human colon (Shoaie et al., 2015), which might explain the depletion of SCFA-producing enzymes in hypertensive gut microbiome. Functionally, SCFAs modulates the gut inflammation and metabolism via functioning as important colonocytes energy source and signaling molecules (Donohoe et al., 2011), suggesting that low level of SCFA production in gut microbiota may be a considerable risk factor of multiple metabolic syndromes and hypertension.

Several other bacteria also played important function in human gut and showed potential function in hypertension, such as, the patient-enriched *Bacteroides* (including *B. eggerthii, B. cellulosilyticus*, and 3 unclassified *Bacteroides* MLGs) and *Parabacteroides* (*P. merdae*) which are generally opportunistic pathogens in infectious diseases and are able to develop antimicrobial drug resistance (Boente et al., 2010), and the control-enriched *Megasphaera* spp. (*M. micronuciformis* and two unclassified MLGs) which are producer of SCFAs, vitamins and essential amino acids (Shetty et al., 2013). In addition, co-abundance analysis (Figure 2A) generated a striking number of positive correlations within the patient/control-enriched MLGs and negative correlations between the two groups, revealing that a comprehensive bacterial synergism and antagonism existed in the human gut. In this case, the microbial dysbiosis of hypertensive gut microbiome would not be determined by independent pathogens (e.g., the patient-enriched MLGs), but more likely to be caused by a series of risk factors (e.g., improper diet or lifestyle that inhibit the growth of beneficial bacterium) that change the balance of ecosystem. Intriguingly, the severity of hypertension was positively correlated with the total abundance of patient-enriched MLGs and negatively correlated with those of control-enriched MLGs (Figure 2B), suggesting that the bacterial relative abundance may also be a potential risk factor of hypertension development. Such a “dose response” was also found in the gut microbiome of liver cirrhosis (Qin et al., 2014) and colorectal adenoma-carcinoma patients (Feng et al., 2015).

Our study further provided the microbial markers for hypertension discrimination, and achieved an AUC of 0.78 for identifying disease status based on 68 species-level MLGs. This discriminatory power was higher than that from the prediction models based on genomic markers identified by GWAS (Evans et al., 2009; Fava et al., 2013), and was almost at same level with the phenotype-based models (AUC 0.71-0.81) (Echouffo-Tcheugui et al., 2013; Wang A. et al., 2015). Thus, the fecal microbiota showed a good potential on prediction and early diagnosis of hypertension, however, systematic investigations of key species and gene markers identified here might be helpful in the future.

Drug-induced gut microbiome shifts were observed during the treatment of multiple diseases, such as, the metformin therapy in type 2 diabetes (Forslund et al., 2015) and antirheumatic drugs therapy in rheumatoid arthritis (Zhang et al., 2015). In this study, a part of patients (~35%) had taken antihypertensive drugs or specific nutritious supplementary, however, the relationship between drug treatment and gut microbiota is still unclear. Another significant limitation of this study is that the gut microbial community would be sensitive to environmental factors, such as, host race, geography, life and diet style, and so on. Although our samples were age-, gender-, BMI-matched, some phenotype differences were still unobservable. To avoid this, larger cohort containing multi-types of hypertensive patients are needed for further investigation. Generally, hypertension is a highly complex and heterogeneous disease, it is still infeasible at this moment to draw any conclusions about causal relationships of gut microbiota and hypertension, and direct experimental studies (e.g., the animal model studies) are needed to show causality of proposed microbes or pathways.

In summary, our finding extends previous knowledge of correlation between gut microbiota and hypertension in animal models (Yang et al., 2015; Durgan et al., 2016) and provides a range of signatures in metagenomic diversity, genes, species, and functions of the hypertensive gut microbiome. Further studies on the causality relationship between hypertension and gut microbiota will lead to a better understanding of the mutual interaction.

## Author contributions

YM, SL, and QY designed experiments; QY, LJ, CC, XH, YH, LZ, PL, and MK carried out experiments; QY, WY, YD, SW, and YX analyzed experimental results. SL, YG, XL, ZF, and JZ analyzed sequencing data. WY, XG, YD, and SW collected the samples. YM, SL, and QY wrote the manuscript.

### Conflict of interest statement

The authors declare that the research was conducted in the absence of any commercial or financial relationships that could be construed as a potential conflict of interest.
