# Supplementary material for: Alterations of the Gut Microbiome in Hypertension
Source: Front Cell Infect Microbiol. 2017 Aug 24;7:381. doi: 10.3389/fcimb.2017.00381 (PMC5573791; doi:10.3389/fcimb.2017.00381)
Supplement: Supplementary file 1 [file DataSheet1.docx]

**Supplementary Methods**

**Clinical parameters measurements**

All clinical parameters were determined by standard procedures. Using a mercury sphygmomanometer, three readings were recorded with a 5-min resting period and the average was taken as the final measurement. To detect FGB, blood samples were drawn following a fasting period of 12 h.

**DNA preparation**

Total bacterial genomic DNA were extracted from about 220 mg of feces using the QIAamp DNA Stool Mini Kit (Qiagen) following the manufacturer’s instruction. Briefly, ASL buffer (1.4 ml) were added to fecal sample and homogenized the pellets in a 2ml screw cap tubes (Axygen) by vortex. The suspension incubated at 95 °C for 5 min to lyse bacterial cells. After samples were put under vortex movement (15s) and centrifugation (13,000×g, 1 min), 1.2ml of supernatants were transferred to fresh 2.0 ml tubes. Then, removal of potential inhibitors by incubation with an InhibitEx tablet, centrifuged InhibitEX matrix (13,000×g, 3 min) were discarded. The supernatant was treated with 15µl proteinase K and 200µl Buffer AL at 70 ℃ for 10 min to remove protein and polysaccharides. DNA was precipitated by 200µl ethanol (96-100%), applied to a column provided in the kit followed by washes with 500µl buffers AW1 and 500µl buffers AW2, and then dissolved in 100µl sterile water. The extracted DNA of each sample was kept frozen at −20 ℃ until used.

**Metagenomic sequencing and data quality control**

Illumina HiSeq 3000 were used to sequence the samples and the libraries were prepared manually following the manufacturer’s protocol (Illumina, USA). Briefly, the DNA samples were normalized to 1.5 μg, and DNA was sheared by sonication with a Covaris LE220. After removed the large DNA fragments (≥650bp) and small DNA fragments (≤250bp), we purified the supernatant using AMPure XP bead (Beckman Coulter, A63880l). Adapters were ligated using 400 units/uL NEB T4 DNA ligase (New England Biolabs, M0202L). Samples were amplified with an 18-cycle PCR using the Kapa HiFi HotStart PCR kit and cleaned up with AMPure beads. A 150 bp paired-end library with insert size of 350 bp was constructed for every sample. The raw sequencing reads for each sample were independently processed quality control using FASTAX Toolkit (http://hannonlab.cshl.edu/fastx_toolkit/). The quality control used the following criteria: (1) reads were removed if it contains more than 3 ‘N’ bases or more than 50 bases with low quality (<Q20); (2) no more than 10 bases with low quality (<Q20) or assigned as N in the tail of reads were trimmed. The remaining reads were then mapped to human genomes using SOAPalinger2[1] to remove the host DNA contaminations. After all, average 0.9% of low-quality or human genome reads were removed for all sequenced samples.

**Taxonomic assignment of MLGs**

As defined in the original paper (Qin et al., 2012; Nielsen et al., 2014), MLG is a group of genes with consistent abundance level (co-abundance) in multiple metagenomic samples which generally referred as microbial species (or named metagenomic species), viruses or other genetic entities. However, due to the lack of comprehensive genomic data of microbes, only a low proportion of metagenomic genes could be reliably assigned to genus (in the human gut gene catalogue, 16.3%) or species (9.7%). In our study, we determined the taxonomic assignment of a MLG by the following principles: 1) if >90% of genes in this MLG can be mapped onto a reference bacterial species with a threshold of 90% nucleotide identity, we assigned the MLG to species assignment; 2) if >40% of genes in this MLG can be mapped onto a reference species with a threshold of 85% identity at the both nucleotide and protein levels, we assigned the MLG to originate from the same genus of the matched species. 50% (34/68) MLGs are classified into species, 19% (13/68) are classified into genus, and the other unclassified MLGs are unknown organisms (Supplement Table 2).

Reference:

Qin et al. A metagenome-wide association study of gut microbiota in type 2 diabetes. Nature. 2012; 490:55-60.

Nielsen et al., Identification and assembly of genomes and genetic elements in complex metagenomic samples without using reference genomes. Nature Biotechnology. 2014; 32:822-828.

**Supplementary Tables**

**Table S1 | Detailed phenotype information of 60 primary hypertensive patients and 60 gender-, age- and BMI-matched heathy controls.**

| **Sample ID** | **Sex** | **Age (y)** | **Height (cm)** | **Weight (kg)** | **BMI (kg/m2)** | **Current SBP (mmHg)** | **Current DBP (mmHg)** | **HT stage*** | **Disease duration (y)** | **FGB (mmol/L)** | **HDL (mmol/L)** | **LDL (mmol/L)** | **TG (mmol/L)** | **TC (mmol/L)** | **Smoke (Y/N)** | **T2D (Y/D)** | **Gene count** | **Shannon index** |
| --- | --- | --- | --- | --- | --- | --- | --- | --- | --- | --- | --- | --- | --- | --- | --- | --- | --- | --- |
| D01 | M | 44 | 174 | 78 | 25.8 | 140 | 86 | 1 | 5 | 8.78 | 1.08 | 3.21 | 1.75 | 4.89 | N | Y | 433020 | 11.6 |
| D02 | F | 65 | 170 | 58 | 20.1 | 150 | 90 | 1 | 0 | 7.33 | 1.28 | 3.94 | 2.02 | 4.94 | N | Y | 562822 | 11.8 |
| D03 | F | 59 | 162 | 65 | 24.8 | 159 | 94 | 1 | 0 | 5.81 | 1.28 | 3.74 | 1.36 | 5.78 | N | N | 620276 | 12 |
| D04 | F | 58 | 155 | 62 | 25.8 | 200 | 130 | 3 | 5 | 9.80 | 1.12 | 3.79 | 2.73 | 5.53 | Y | Y | 585262 | 11.4 |
| D05 | M | 47 | 169 | 52 | 18.2 | 150 | 92 | 1 | 6 | 5.83 | 1.16 | 2.94 | 2.00 | 4.11 | N | N | 442627 | 11.5 |
| D06 | M | 52 | 165 | 64 | 23.5 | 207 | 122 | 3 | 3 | 5.46 | 0.94 | 3.44 | 1.76 | 4.76 | Y | N | 266509 | 11.1 |
| D07 | M | 63 | 168 | 71 | 25.2 | 145 | 95 | 1 | 1 | 4.97 | 0.82 | 4.31 | 2.65 | 6.01 | N | N | 470121 | 11.5 |
| D08 | M | 46 | 175 | 63 | 20.6 | 145 | 90 | 1 | 0 | 4.27 | 1.12 | 2.24 | 0.84 | 4.63 | N | N | 198327 | 10.6 |
| D09 | M | 45 | 154 | 60 | 25.3 | 166 | 104 | 2 | 3 | 12.50 | 0.96 | 2.98 | 2.73 | 4.53 | N | Y | 286774 | 11.3 |
| D10 | F | 62 | 162 | 64 | 24.4 | 173 | 105 | 2 | 1 | 14.35 | 0.90 | 3.74 | 2.92 | 5.60 | N | Y | 380153 | 11.6 |
| D11 | M | 73 | 168 | 61 | 21.6 | 176 | 103 | 2 | 0 | 4.22 | 1.31 | 2.20 | 1.87 | 3.85 | Y | N | 349923 | 11 |
| D12 | M | 73 | 169 | 76 | 26.6 | 161 | 97 | 2 | 0 | 5.05 | 1.05 | 3.22 | 2.05 | 5.22 | N | N | 521713 | 10.6 |
| D13 | M | 65 | 158 | 65 | 26 | 150 | 95 | 1 | 3 | 7.59 | 1.21 | 3.19 | 2.62 | 4.97 | N | Y | 187807 | 10.8 |
| D14 | F | 64 | 151 | 73 | 32 | 145 | 90 | 1 | 4 | 10.68 | 1.19 | 2.45 | 4.23 | 4.67 | N | Y | 446235 | 10.2 |
| D15 | M | 49 | 167 | 67 | 24 | 190 | 110 | 3 | 5 | 4.91 | 1.13 | 2.85 | 1.50 | 4.58 | N | N | 191092 | 11 |
| D16 | M | 61 | 163 | 55 | 20.7 | 140 | 90 | 1 | 0 | 5.09 | 1.07 | 2.91 | 1.31 | 4.47 | N | N | 495358 | 11.5 |
| D17 | M | 47 | 162 | 65 | 24.8 | 152 | 95 | 1 | 3 | 4.53 | 0.70 | 2.87 | 1.12 | 4.56 | N | N | 485258 | 11.8 |
| D18 | M | 55 | 164 | 52 | 19.3 | 140 | 90 | 1 | 2 | 8.71 | 1.59 | 2.55 | 3.71 | 6.24 | N | Y | 665340 | 12 |
| D19 | M | 49 | 162 | 54 | 20.6 | 182 | 107 | 3 | 6 | 4.74 | 1.54 | 2.95 | 3.31 | 4.95 | Y | N | 190347 | 10.9 |
| D20 | F | 59 | 155 | 61 | 25.4 | 160 | 100 | 2 | 7 | 8.62 | 1.18 | 3.02 | 3.35 | 4.80 | N | Y | 238327 | 11 |
| D21 | F | 55 | 170 | 73 | 25.3 | 180 | 95 | 3 | 5 | 4.33 | 1.28 | 2.55 | 0.87 | 5.74 | N | N | 345304 | 10.8 |
| D22 | F | 46 | 168 | 64 | 22.7 | 175 | 102 | 2 | 3 | 4.67 | 1.59 | 2.81 | 1.42 | 5.27 | N | N | 495705 | 11.9 |
| D23 | F | 51 | 155 | 50 | 20.8 | 180 | 110 | 3 | 6 | 4.65 | 1.36 | 1.72 | 0.71 | 3.36 | Y | N | 177456 | 10.9 |
| D24 | M | 45 | 153 | 59 | 25.2 | 160 | 90 | 2 | 5 | 8.32 | 1.16 | 2.62 | 0.76 | 4.16 | N | Y | 508633 | 11.8 |
| D25 | F | 62 | 166 | 70 | 25.4 | 150 | 90 | 1 | 2 | 5.06 | 0.92 | 3.26 | 0.45 | 5.88 | N | N | 464462 | 11.7 |
| D26 | F | 74 | 161 | 59 | 22.8 | 190 | 124 | 3 | 0 | 4.50 | 1.20 | 2.21 | 0.88 | 4.17 | Y | N | 415245 | 11 |
| D27 | F | 43 | 160 | 66 | 25.8 | 170 | 95 | 2 | 1 | 8.30 | 1.07 | 2.31 | 2.45 | 4.65 | N | Y | 329338 | 11.6 |
| D28 | M | 48 | 157 | 45 | 18.3 | 140 | 90 | 1 | 0 | 5.51 | 1.48 | 4.81 | 1.97 | 7.04 | Y | N | 362556 | 11.3 |
| D29 | M | 51 | 157 | 66 | 26.8 | 210 | 121 | 3 | 4 | 5.02 | 1.29 | 3.26 | 0.81 | 4.75 | N | N | 335551 | 11.3 |
| D30 | M | 58 | 161 | 72 | 27.8 | 141 | 90 | 1 | 0 | 5.66 | 1.24 | 3.07 | 1.32 | 5.46 | N | N | 357520 | 10.9 |
| D31 | M | 61 | 165 | 56 | 20.6 | 194 | 100 | 3 | 3 | 4.82 | 1.71 | 3.36 | 1.29 | 5.56 | N | N | 261185 | 11 |
| D32 | M | 54 | 173 | 76 | 25.4 | 145 | 90 | 1 | 4 | 4.79 | 0.89 | 2.37 | 0.97 | 4.21 | N | N | 517583 | 12 |
| D33 | M | 72 | 159 | 51 | 20.2 | 140 | 90 | 1 | 0 | 11.09 | 0.88 | 5.26 | 3.52 | 8.73 | N | Y | 327577 | 10.9 |
| D34 | M | 62 | 152 | 50 | 21.6 | 165 | 95 | 2 | 0 | 4.50 | 1.14 | 2.46 | 1.03 | 4.33 | N | N | 381033 | 10.1 |
| D35 | M | 54 | 163 | 68 | 25.6 | 195 | 121 | 3 | 1 | 5.60 | 1.49 | 2.95 | 1.37 | 5.54 | Y | N | 327843 | 10.8 |
| D36 | F | 52 | 169 | 58 | 20.3 | 185 | 105 | 3 | 2 | 5.65 | 1.45 | 3.32 | 1.13 | 4.98 | N | N | 514075 | 11.6 |
| D37 | F | 68 | 152 | 51 | 22.1 | 165 | 100 | 2 | 0 | 5.99 | 1.10 | 4.15 | 1.20 | 5.94 | N | N | 513191 | 11.4 |
| D38 | F | 62 | 180 | 70 | 21.6 | 145 | 100 | 1 | 6 | 6.78 | 0.81 | 4.11 | 2.55 | 5.70 | N | Y | 277070 | 10.9 |
| D39 | M | 44 | 169 | 72 | 25.2 | 149 | 98 | 1 | 0 | 4.90 | 0.62 | 1.79 | 2.23 | 3.00 | Y | N | 346644 | 10.2 |
| D40 | F | 68 | 175 | 80 | 26.1 | 195 | 115 | 3 | 0 | 7.93 | 1.03 | 3.95 | 1.76 | 5.53 | Y | Y | 394119 | 11.1 |
| D41 | M | 75 | 160 | 75 | 29.3 | 170 | 110 | 2 | 2 | 5.71 | 0.97 | 2.81 | 1.59 | 5.10 | N | N | 269388 | 11.1 |
| D42 | M | 50 | 172 | 70 | 23.7 | 180 | 100 | 3 | 8 | 5.14 | 1.49 | 3.64 | 1.26 | 3.98 | Y | N | 539928 | 11.2 |
| D43 | M | 46 | 164 | 55 | 20.4 | 165 | 101 | 2 | 0 | 6.00 | 0.65 | 1.38 | 1.23 | 2.68 | Y | N | 775189 | 12.3 |
| D44 | F | 54 | 170 | 63 | 21.8 | 161 | 107 | 2 | 4 | 4.73 | 1.31 | 3.43 | 1.79 | 5.47 | Y | N | 443474 | 11.7 |
| D45 | M | 58 | 170 | 55 | 19 | 140 | 85 | 1 | 1 | 5.73 | 1.30 | 3.16 | 0.98 | 5.44 | Y | N | 419971 | 11.3 |
| D46 | F | 70 | 158 | 59 | 23.6 | 165 | 92 | 2 | 0 | 3.13 | 1.03 | 2.45 | 1.20 | 5.27 | N | N | 463079 | 10.6 |
| D47 | F | 57 | 165 | 56 | 20.6 | 192 | 105 | 3 | 10 | 5.00 | 1.51 | 4.26 | 2.44 | 5.84 | N | N | 162971 | 10.4 |
| D48 | F | 47 | 153 | 56 | 23.9 | 200 | 110 | 3 | 2 | 4.66 | 1.02 | 3.31 | 1.44 | 4.94 | N | N | 172407 | 9.7 |
| D49 | F | 74 | 165 | 54 | 19.8 | 160 | 95 | 2 | 3 | 5.70 | 1.09 | 2.38 | 0.96 | 4.92 | N | N | 211181 | 10.4 |
| D50 | F | 45 | 155 | 60 | 25 | 140 | 90 | 1 | 0 | 9.52 | 1.25 | 3.13 | 2.51 | 5.25 | N | Y | 495286 | 11.8 |
| D51 | M | 49 | 170 | 65 | 22.5 | 180 | 124 | 3 | 4 | 5.61 | 1.37 | 3.53 | 1.36 | 5.54 | N | N | 468325 | 11.5 |
| D52 | M | 72 | 163 | 68 | 25.6 | 170 | 100 | 2 | 3 | 4.61 | 1.37 | 4.35 | 1.73 | 6.92 | Y | N | 474266 | 11.6 |
| D53 | F | 76 | 161 | 50 | 19.3 | 145 | 90 | 1 | 0 | 4.19 | 1.04 | 2.76 | 2.11 | 4.25 | N | N | 665373 | 12.2 |
| D54 | M | 64 | 156 | 66 | 27.1 | 145 | 95 | 1 | 0 | 12.93 | 0.96 | 3.15 | 2.80 | 5.29 | Y | Y | 425316 | 11.9 |
| D55 | F | 54 | 170 | 64 | 22.1 | 167 | 105 | 2 | 5 | 4.93 | 1.46 | 1.76 | 1.88 | 3.20 | Y | N | 358671 | 10.8 |
| D56 | M | 63 | 158 | 66 | 26.4 | 189 | 117 | 3 | 10 | 5.90 | 0.75 | 3.02 | 2.44 | 5.67 | N | Y | 398502 | 11.4 |
| D57 | F | 48 | 171 | 67 | 22.9 | 180 | 113 | 3 | 1 | 4.95 | 1.08 | 2.33 | 1.21 | 4.27 | N | N | 748853 | 12 |
| D58 | M | 49 | 160 | 68 | 26.6 | 160 | 98 | 2 | 2 | 7.93 | 0.96 | 3.06 | 3.01 | 4.23 | Y | Y | 256559 | 11.2 |
| D59 | M | 47 | 171 | 74 | 25.3 | 160 | 115 | 2 | 0 | 9.38 | 0.92 | 2.35 | 2.83 | 4.89 | Y | Y | 201258 | 10.9 |
| D60 | M | 57 | 171 | 64 | 21.9 | 140 | 90 | 1 | 0 | 5.43 | 1.15 | 3.60 | 1.47 | 5.01 | N | N | 313206 | 11 |
| H01 | F | 54 | 162 | 61 | 23.2 | 92 | 61 | - | - | 5.42 | 1.17 | 2.36 | 1.04 | 3.67 | N | N | 795429 | 12.4 |
| H02 | F | 57 | 158 | 62 | 24.8 | 120 | 78 | - | - | 6.60 | 1.31 | 2.28 | 1.69 | 4.12 | N | Y | 764588 | 12.3 |
| H03 | F | 48 | 168 | 58 | 20.5 | 110 | 70 | - | - | 5.41 | 0.89 | 2.89 | 1.99 | 5.06 | Y | N | 551418 | 11.9 |
| H04 | M | 54 | 155 | 65 | 27.1 | 117 | 73 | - | - | 5.05 | 1.20 | 3.07 | 0.68 | 4.40 | N | N | 392312 | 10.9 |
| H05 | M | 64 | 154 | 50 | 21.1 | 116 | 60 | - | - | 10.12 | 1.05 | 2.97 | 2.03 | 7.50 | N | Y | 597828 | 12.1 |
| H06 | F | 63 | 155 | 44 | 18.3 | 110 | 60 | - | - | 8.94 | 1.24 | 2.32 | 1.43 | 3.24 | N | Y | 610566 | 12.1 |
| H07 | F | 46 | 147 | 52 | 24.1 | 112 | 80 | - | - | 5.61 | 1.31 | 3.04 | 1.12 | 5.12 | N | N | 388157 | 10.6 |
| H08 | F | 57 | 165 | 60 | 22 | 110 | 60 | - | - | 9.20 | 1.88 | 2.87 | 1.30 | 5.83 | N | Y | 243253 | 11.3 |
| H09 | M | 67 | 160 | 68 | 26.6 | 100 | 70 | - | - | 4.32 | 0.90 | 2.37 | 2.58 | 4.99 | Y | N | 307076 | 11.3 |
| H10 | M | 44 | 148 | 43 | 25 | 110 | 70 | - | - | 5.70 | 1.00 | 2.45 | 1.31 | 3.70 | Y | N | 575115 | 12.3 |
| H11 | M | 58 | 170 | 61 | 21.1 | 102 | 61 | - | - | 3.90 | 1.35 | 2.54 | 0.60 | 3.94 | Y | N | 524984 | 11.5 |
| H12 | F | 71 | 155 | 64 | 26.6 | 118 | 65 | - | - | 4.76 | 0.76 | 2.08 | 1.79 | 3.79 | Y | N | 460925 | 11.7 |
| H13 | F | 50 | 158 | 48 | 19.2 | 120 | 79 | - | - | 4.34 | 1.45 | 2.36 | 0.42 | 4.32 | N | N | 727875 | 12.6 |
| H14 | M | 70 | 170 | 67 | 23.2 | 115 | 64 | - | - | 4.92 | 1.48 | 3.12 | 0.65 | 5.58 | N | N | 562456 | 11.8 |
| H15 | M | 72 | 173 | 65 | 21.7 | 98 | 68 | - | - | 5.29 | 1.10 | 3.03 | 1.02 | 4.67 | Y | N | 767510 | 11.8 |
| H16 | F | 72 | 151 | 54 | 23.7 | 110 | 73 | - | - | 12.38 | 1.29 | 4.11 | 1.88 | 5.93 | N | Y | 228425 | 11.1 |
| H17 | F | 51 | 170 | 71 | 24.6 | 110 | 67 | - | - | 5.23 | 1.13 | 2.90 | 0.68 | 4.76 | N | N | 459154 | 10.6 |
| H18 | M | 46 | 157 | 60 | 24.3 | 110 | 80 | - | - | 4.23 | 1.15 | 3.83 | 0.84 | 4.82 | Y | N | 193686 | 10.2 |
| H19 | M | 50 | 160 | 63 | 24.6 | 120 | 75 | - | - | 5.32 | 1.05 | 3.04 | 1.50 | 4.32 | Y | N | 484963 | 11.8 |
| H20 | F | 69 | 160 | 55 | 21.5 | 115 | 65 | - | - | 5.05 | 1.16 | 4.14 | 1.49 | 6.16 | N | N | 396879 | 11.7 |
| H21 | M | 68 | 168 | 59 | 20.9 | 115 | 75 | - | - | 8.39 | 0.99 | 2.66 | 1.48 | 6.48 | Y | Y | 554767 | 11.8 |
| H22 | M | 62 | 165 | 50 | 18.4 | 110 | 70 | - | - | 6.99 | 1.11 | 2.56 | 2.31 | 4.90 | N | Y | 674720 | 12.7 |
| H23 | M | 42 | 167 | 75 | 26.9 | 114 | 82 | - | - | 7.07 | 1.30 | 3.83 | 2.04 | 4.72 | Y | Y | 360744 | 11.2 |
| H24 | M | 60 | 169 | 64 | 22.4 | 116 | 80 | - | - | 5.66 | 1.28 | 3.22 | 0.93 | 5.25 | N | N | 298113 | 10.6 |
| H25 | F | 43 | 164 | 58 | 21.6 | 119 | 79 | - | - | 4.92 | 0.96 | 3.42 | 2.08 | 5.65 | Y | N | 311518 | 11.7 |
| H26 | F | 55 | 148 | 52 | 23.7 | 105 | 65 | - | - | 4.38 | 1.12 | 5.15 | 2.24 | 7.13 | N | N | 340261 | 10.8 |
| H27 | F | 57 | 162 | 56 | 21.3 | 112 | 75 | - | - | 4.94 | 1.09 | 3.82 | 1.53 | 5.93 | N | N | 669101 | 12 |
| H28 | M | 50 | 172 | 80 | 27 | 120 | 76 | - | - | 4.96 | 1.94 | 4.39 | 0.80 | 7.07 | N | N | 472194 | 11.7 |
| H29 | M | 53 | 160 | 45 | 17.6 | 112 | 80 | - | - | 8.90 | 0.98 | 2.88 | 2.30 | 3.96 | Y | Y | 404874 | 11.6 |
| H30 | M | 65 | 164 | 62 | 23.1 | 110 | 80 | - | - | 6.68 | 1.38 | 2.17 | 0.63 | 3.70 | N | Y | 470943 | 11.2 |
| H31 | M | 54 | 166 | 68 | 24.7 | 118 | 72 | - | - | 8.30 | 1.17 | 2.64 | 1.37 | 4.13 | N | Y | 294732 | 10.2 |
| H32 | F | 71 | 168 | 78 | 27.6 | 110 | 83 | - | - | 4.85 | 1.31 | 3.13 | 1.26 | 5.51 | N | N | 507792 | 11 |
| H33 | F | 46 | 172 | 75 | 25.4 | 111 | 66 | - | - | 4.26 | 1.28 | 2.12 | 1.11 | 6.13 | N | N | 371245 | 11.2 |
| H34 | F | 64 | 172 | 73 | 24.7 | 108 | 64 | - | - | 8.50 | 1.20 | 2.03 | 0.69 | 3.94 | N | Y | 641375 | 11.9 |
| H35 | M | 45 | 150 | 50 | 22.2 | 112 | 72 | - | - | 9.11 | 1.46 | 2.59 | 2.50 | 4.81 | Y | Y | 324984 | 11.3 |
| H36 | M | 56 | 155 | 50 | 20.8 | 110 | 70 | - | - | 7.83 | 0.99 | 4.03 | 2.35 | 5.31 | Y | Y | 446405 | 11.8 |
| H37 | M | 56 | 156 | 60 | 24.9 | 107 | 63 | - | - | 5.62 | 0.96 | 2.43 | 2.00 | 5.04 | N | N | 493551 | 10.6 |
| H38 | F | 43 | 153 | 54 | 23.1 | 118 | 74 | - | - | 5.05 | 1.61 | 4.14 | 1.96 | 5.99 | N | N | 378477 | 11.4 |
| H39 | M | 53 | 178 | 78 | 24.6 | 109 | 66 | - | - | 4.27 | 1.44 | 2.52 | 0.97 | 4.58 | Y | N | 475321 | 11.5 |
| H40 | M | 62 | 160 | 59 | 23 | 94 | 61 | - | - | 7.20 | 1.33 | 2.41 | 0.56 | 4.03 | N | Y | 410302 | 11.6 |
| H41 | F | 56 | 158 | 74 | 29.6 | 109 | 75 | - | - | 6.96 | 1.74 | 3.52 | 2.26 | 4.49 | N | Y | 298536 | 10.9 |
| H42 | F | 58 | 155 | 46 | 19.1 | 120 | 70 | - | - | 8.28 | 1.03 | 3.83 | 2.62 | 5.65 | Y | Y | 450074 | 11.7 |
| H43 | M | 58 | 163 | 54 | 20.3 | 116 | 78 | - | - | 4.23 | 2.02 | 2.25 | 0.45 | 4.95 | Y | N | 455182 | 11.8 |
| H44 | M | 54 | 150 | 50 | 22.2 | 110 | 76 | - | - | 5.16 | 1.93 | 4.14 | 1.13 | 6.21 | Y | N | 269548 | 11.2 |
| H45 | F | 60 | 164 | 68 | 25.3 | 112 | 80 | - | - | 4.48 | 1.43 | 2.29 | 0.96 | 4.13 | N | N | 421622 | 11.6 |
| H46 | F | 50 | 150 | 48 | 21.3 | 112 | 68 | - | - | 4.23 | 0.97 | 2.75 | 1.61 | 4.06 | N | N | 640936 | 11.9 |
| H47 | M | 46 | 170 | 62 | 21.5 | 110 | 70 | - | - | 14.06 | 1.00 | 3.05 | 2.08 | 5.44 | Y | Y | 706102 | 11.9 |
| H48 | F | 43 | 175 | 72 | 23.5 | 107 | 80 | - | - | 5.02 | 0.72 | 2.83 | 1.26 | 3.59 | N | N | 524475 | 12.1 |
| H49 | F | 59 | 165 | 71 | 26.1 | 110 | 70 | - | - | 9.72 | 1.01 | 3.95 | 2.18 | 6.57 | N | Y | 660846 | 12 |
| H50 | M | 48 | 160 | 63 | 24.6 | 116 | 69 | - | - | 4.78 | 1.25 | 2.50 | 1.75 | 4.29 | Y | N | 342181 | 11.1 |
| H51 | F | 55 | 150 | 58 | 25.8 | 105 | 80 | - | - | 5.51 | 0.92 | 3.91 | 2.12 | 5.24 | Y | N | 315899 | 10.7 |
| H52 | M | 71 | 171 | 74 | 25.3 | 110 | 80 | - | - | 5.69 | 1.45 | 2.69 | 0.59 | 5.75 | N | N | 629653 | 11.9 |
| H53 | M | 49 | 180 | 90 | 27.8 | 110 | 70 | - | - | 7.73 | 0.77 | 3.90 | 3.66 | 6.01 | Y | Y | 418663 | 11.1 |
| H54 | F | 51 | 168 | 75 | 26.6 | 115 | 70 | - | - | 5.12 | 1.31 | 3.06 | 1.72 | 4.63 | N | N | 378294 | 11.1 |
| H55 | M | 55 | 167 | 55 | 19.7 | 106 | 72 | - | - | 5.29 | 1.02 | 2.06 | 2.43 | 6.01 | N | N | 363596 | 11.3 |
| H56 | F | 46 | 150 | 50 | 22.2 | 112 | 62 | - | - | 5.53 | 1.47 | 2.79 | 1.47 | 6.40 | N | N | 703268 | 12.3 |
| H57 | F | 48 | 152 | 51 | 22.1 | 118 | 66 | - | - | 5.50 | 1.07 | 3.70 | 1.75 | 5.67 | N | N | 602223 | 12.1 |
| H58 | M | 60 | 168 | 71 | 25.2 | 110 | 60 | - | - | 4.74 | 0.99 | 3.29 | 2.35 | 5.21 | N | N | 215491 | 10.5 |
| H59 | M | 52 | 168 | 65 | 23 | 95 | 65 | - | - | 5.18 | 1.26 | 3.61 | 0.81 | 5.17 | Y | N | 333237 | 11.4 |
| H60 | M | 70 | 158 | 68 | 27.2 | 110 | 80 | - | - | 4.40 | 1.57 | 2.15 | 0.78 | 4.43 | Y | N | 392164 | 11.7 |

*Stage I: current blood pressure 140/90 ~ 159/99; stage II: current blood pressure 160/100 ~ 179~109; stage III: current blood pressure >180/110.

**Table S2 | Detailed information of 68 hypertension-associated MLGs.**

| MLG ID | Enriched | No. of genes | P-value | Taxonomy assignment | Best BLASTN to NCBI genomes | % gene matched | % similarity |
| --- | --- | --- | --- | --- | --- | --- | --- |
| HT-01 | patient | 4364 | 0.03322003 | *Klebsiella pneumoniae* | *Klebsiella pneumoniae* | 92.2 | 97.5 |
| HT-02 | patient | 2614 | 0.01711132 | *Sutterella wadsworthensis* | *Sutterella wadsworthensis* | 94.9 | 92.4 |
| HT-03 | patient | 1711 | 0.00908569 | *Parabacteroides merdae* | *Parabacteroides merdae* | 98.9 | 94.2 |
| HT-04 | patient | 1239 | 0.00822939 | *Klebsiella variicola* | *Klebsiella variicola* | 97.7 | 97.3 |
| HT-05 | patient | 1045 | 0.01510564 | *Pyramidobacter piscolens* | *Pyramidobacter piscolens* | 94.3 | 99.3 |
| HT-06 | patient | 899 | 0.01293128 | *Mitsuokella multacida* | *Mitsuokella multacida* | 95.6 | 95.6 |
| HT-07 | patient | 580 | 0.00417114 | *Streptococcus infantarius* | *Streptococcus infantarius* | 97.4 | 97 |
| HT-08 | patient | 522 | 0.00563052 | *Bacteroides* (genus) *** | *Bacteroides nordii* | 40.3 | 92.4 |
| HT-09 | patient | 494 | 0.01651744 | *Streptococcus* (genus) * | *Streptococcus vestibularis* | 52.9 | 86.3 |
| HT-10 | patient | 389 | 0.01409845 | *Bacteroides* (genus) * | *Bacteroides coprocola* | 49.9 | 90.6 |
| HT-11 | patient | 331 | 0.00831803 | *Butyricimonas* (genus) * | *Butyricimonas synergistica* | 56.3 | 90.7 |
| HT-12 | patient | 327 | 0.00672481 | *Dorea longicatena* | *Dorea longicatena* | 98.5 | 95.9 |
| HT-13 | patient | 312 | 0.03891346 | *Klebsiella* (genus) * | *Klebsiella variicola* | 41.7 | 92.9 |
| HT-14 | patient | 310 | 0.02809764 | *Streptococcus pasteurianus* | *Streptococcus pasteurianus* | 91.6 | 96.9 |
| HT-15 | patient | 255 | 0.00533973 | *Bacteroides eggerthii* | *Bacteroides eggerthii* | 97.6 | 94.4 |
| HT-16 | patient | 202 | 0.00261792 | *Streptococcus salivarius* | *Streptococcus salivarius* | 88.9 | 94.2 |
| HT-17 | patient | 187 | 0.0440725 | *Klebsiella* (genus) * | *Klebsiella pneumoniae* | 33.2 | 93.2 |
| HT-18 | patient | 167 | 0.02089866 | *Eggerthella lenta* | *Eggerthella lenta* | 87.1 | 97.9 |
| HT-19 | patient | 159 | 0.03138614 | *Adlercreutzia equolifaciens* | *Adlercreutzia equolifaciens* | 97.5 | 93.9 |
| HT-20 | patient | 151 | 0.01493045 | *Prevotella* (genus) * | *Prevotella copri* | 45.8 | 90.4 |
| HT-21 | patient | 118 | 0.01135578 | *Bifidobacterium* (genus) * | *Bifidobacterium adolescentis* | 56.8 | 85.6 |
| HT-22 | patient | 104 | 0.03328649 | *Bacteroides cellulosilyticus* | *Bacteroides cellulosilyticus* | 91.3 | 90.1 |
| HT-23 | patient | 798 | 0.03062621 | Unclassified | *Flavonifractor plautii* | 5.9 | 72.6 |
| HT-24 | patient | 655 | 0.01234328 | Unclassified | *Senegalimassilia anaerobia* | 20 | 87.4 |
| HT-25 | patient | 463 | 0.03073094 | Unclassified | *Ruminococcus sp. JC304* | 7.4 | 86.4 |
| HT-26 | patient | 424 | 0.01361464 | *Klebsiella* (genus) ** | *Klebsiella pneumoniae* | 9.3 | 90.3 |
| HT-27 | patient | 336 | 0.02520229 | *Klebsiella* (genus) ** | *Klebsiella variicola* | 17.8 | 75.4 |
| HT-28 | patient | 280 | 0.0072416 | *Streptococcus* (genus) ** | *Streptococcus parasanguinis* | 27.8 | 79.6 |
| HT-29 | patient | 243 | 0.00291503 | Unclassified | *Faecalibacterium prausnitzii* | 8.4 | 73.5 |
| HT-30 | patient | 143 | 0.00892511 | Unclassified | *Streptococcus parasanguinis* | 9.1 | 83.3 |
| HT-31 | patient | 124 | 0.01621042 | Unclassified | *Klebsiella pneumoniae* | 15.3 | 76.3 |
| CON-01 | control | 1635 | 0.0079033 | *Faecalibacterium prausnitzii* | *Faecalibacterium prausnitzii* | 99.5 | 99.7 |
| CON-02 | control | 1276 | 0.02910207 | *Roseburia intestinalis* | *Roseburia intestinalis* | 95.5 | 95.2 |
| CON-03 | control | 956 | 0.00487703 | *Aeromicrobium massiliense* | *Aeromicrobium massiliense* | 96.8 | 95.1 |
| CON-04 | control | 741 | 0.00427819 | *Clostridium hathewayi* | *Clostridium hathewayi* | 97.4 | 99.6 |
| CON-05 | control | 645 | 0.00395967 | *Megasphaera micronuciformis* | *Megasphaera micronuciformis* | 97.2 | 91.1 |
| CON-06 | control | 589 | 0.01300351 | *Blautia product* | *Blautia product* | 93.8 | 92.5 |
| CON-07 | control | 586 | 0.00427364 | *Roseburia hominis* | *Roseburia hominis* | 95.5 | 93.5 |
| CON-08 | control | 510 | 0.02179431 | *Intestinibacter bartlettii* | *Intestinibacter bartlettii* | 95.4 | 93.6 |
| CON-09 | control | 464 | 0.00298482 | *Prevotella bivia* | *Prevotella bivia* | 95.2 | 99.6 |
| CON-10 | control | 458 | 0.00088534 | *Megasphaera* (genus) * | *Megasphaera micronuciformis* | 54.3 | 87.1 |
| CON-11 | control | 406 | 0.00132946 | *Bacteroides uniformis* | *Bacteroides uniformis* | 94.6 | 96.3 |
| CON-12 | control | 388 | 0.00059108 | *Prevotella* (genus) * | *Prevotella timonensis* | 40.5 | 88.3 |
| CON-13 | control | 370 | 0.00689193 | *Roseburia intestinalis* | *Roseburia intestinalis* | 91.3 | 98.9 |
| CON-14 | control | 331 | 0.00125069 | *Megasphaera* (genus) * | *Veillonella sp. oral taxon 158* | 54.4 | 87.2 |
| CON-15 | control | 283 | 0.00043801 | *Blautia hansenii* | *Blautia hansenii* | 95.2 | 91.3 |
| CON-16 | control | 281 | 0.01879896 | *Collinsella aerofaciens* | *Collinsella aerofaciens* | 92.6 | 93.4 |
| CON-17 | control | 242 | 0.00119211 | *Faecalitalea* (genus) * | *Faecalitalea cylindroides* | 34 | 90.9 |
| CON-18 | control | 230 | 0.02954748 | *Clostridium citroniae* | *Clostridium citroniae* | 96.7 | 90.4 |
| CON-19 | control | 214 | 0.01071826 | *Bacteroides nordii* | *Bacteroides nordii* | 98.2 | 98.3 |
| CON-20 | control | 197 | 0.00162255 | *Bifidobacterium dentium* | *Bifidobacterium dentium* | 100 | 93.7 |
| CON-21 | control | 147 | 0.01314107 | *Fusobacterium* (genus) * | *Fusobacterium nucleatum* | 40.8 | 88.9 |
| CON-22 | control | 140 | 0.03476184 | *Flavonifractor plautii* | *Flavonifractor plautii* | 95 | 96.2 |
| CON-23 | control | 124 | 0.01920147 | *Bacteroides dorei* | *Bacteroides dorei* | 93.2 | 94.2 |
| CON-24 | control | 114 | 0.02143371 | *Haemophilus parainfluenzae* | *Haemophilus parainfluenzae* | 90.2 | 97 |
| CON-25 | control | 107 | 0.00780934 | *Holdemania filiformis* | *Holdemania filiformis* | 99.1 | 98 |
| CON-26 | control | 961 | 0.01461956 | Unclassified | *Blautia producta* | 1 | 73 |
| CON-27 | control | 814 | 7.50611E-05 | *Clostridiales* (order) ** | *Faecalibacterium prausnitzii* | 8 | 86.7 |
| CON-28 | control | 747 | 0.03275115 | *Clostridiales* (order) ** | *Ruminococcus sp. JC304* | 10.6 | 81.5 |
| CON-29 | control | 732 | 0.00284656 | *Clostridiales* (order) ** | *Flavonifractor plautii* | 24.5 | 73.3 |
| CON-30 | control | 493 | 0.00067044 | Unclassified | *Collinsella aerofaciens* | 7.9 | 74.4 |
| CON-31 | control | 450 | 0.00066334 | Unclassified | *Aeromicrobium massiliense* | 14.3 | 83.1 |
| CON-32 | control | 341 | 0.00096034 | Unclassified | *Streptococcus australis* | 9.4 | 76.3 |
| CON-33 | control | 284 | 0.00192187 | Unclassified | *Pseudoflavonifractor capillosus* | 5.1 | 75.6 |
| CON-34 | control | 270 | 0.01241675 | *Ruminococcus* (genus) ** | *Ruminococcus champanellensis* | 20.9 | 82.3 |
| CON-35 | control | 211 | 0.00071379 | Unclassified | *Veillonella dispar* | 16.1 | 76.4 |
| CON-36 | control | 152 | 0.00078457 | *Bacteroidales* (order) ** | *Prevotella disiens* | 24.1 | 70.6 |
| CON-37 | control | 130 | 0.00248354 | Unclassified | *Prevotella timonensis* | 11.8 | 76 |

*: assigned to taxonomy level at nucleic acid level (>40% genes, >85% identity).

**: assigned to taxonomy level at protein level (>40% genes, >85% identity).

**Table S3 | KEGG pathways that differed significantly between two cohorts.**

| Pathway ID | Average abundance (patient) | Average abundance (control) | P-value | level1 | level2 | level3 |
| --- | --- | --- | --- | --- | --- | --- |
| **patient-enriched** | |  |  |  |  |  |
| ko00540 | 0.00297193 | 0.00228846 | 0.01505778 | M | Glycan biosynthesis and metabolism | Lipopolysaccharide biosynthesis |
| ko00984 | 6.6254E-05 | 3.8779E-05 | 0.00858513 | M | Xenobiotics biodegradation and metabolism | Steroid degradation |
| ko02010 | 0.0210572 | 0.01510195 | 0.00458441 | E | Membrane transport | ABC transporters |
| ko02060 | 0.00336031 | 0.00238542 | 0.00606705 | E | Membrane transport | Phosphotransferase system (PTS) |
| ko03070 | 0.00857131 | 0.00566288 | 0.00638744 | E | Membrane transport | Bacterial secretion system |
| **control-enriched** | |  |  |  |  |  |
| ko00130 | 0.00144524 | 0.0019216 | 0.01794748 | M | Metabolism of cofactors and vitamins | Ubiquinone and other terpenoid-quinone biosynthesis |
| ko00410 | 0.00084869 | 0.00110934 | 0.02054318 | M | Metabolism of other amino acids | beta-Alanine metabolism |
| ko00450 | 0.00381099 | 0.00487369 | 0.0349442 | M | Metabolism of other amino acids | Selenocompound metabolism |
| ko00460 | 0.00434905 | 0.00566285 | 0.01321973 | M | Metabolism of other amino acids | Cyanoamino acid metabolism |
| ko00473 | 0.00102868 | 0.00171665 | 0.0383798 | M | Metabolism of other amino acids | D-Alanine metabolism |
| ko00670 | 0.00436077 | 0.00629146 | 0.00372613 | M | Metabolism of cofactors and vitamins | One carbon pool by folate |
| ko00740 | 0.00143387 | 0.0023633 | 0.01425082 | M | Metabolism of cofactors and vitamins | Riboflavin metabolism |
| ko00790 | 0.00290028 | 0.00377312 | 0.01188197 | M | Metabolism of cofactors and vitamins | Folate biosynthesis |

M: Metabolism; E: Environmental Information Processing
